# Regulation of the Staphylococcal Superantigen-Like Protein 1 Gene of Community-Associated Methicillin-Resistant *Staphylococcus aureus* in Murine Abscesses

**DOI:** 10.3390/toxins11070391

**Published:** 2019-07-04

**Authors:** Daniel J. Bretl, Abdulaziz Elfessi, Hannah Watkins, William R. Schwan

**Affiliations:** 1 Department of Microbiology, University of Wisconsin-La Crosse, La Crosse, WI 54601, USA; 2 Department of Mathematics and Statistics, University of Wisconsin-La Crosse, La Crosse, WI 54601, USA

**Keywords:** mouse abscess, methicillin-resistant *Staphylococcus aureus*, lux fusion, superantigen-like protein, gene regulation, defined minimal medium

## Abstract

Community-associated methicillin-resistant *Staphylococcus aureus* (CA-MRSA) causes substantial skin and soft tissue infections annually in the United States and expresses numerous virulence factors, including a family of toxins known as the staphylococcal superantigen-like (SSL) proteins. Many of the SSL protein structures have been determined and implicated in immune system avoidance, but the full scope that these proteins play in different infection contexts remains unknown and continues to warrant investigation. Analysis of *ssl* gene regulation may provide valuable information related to the function of these proteins. To determine the transcriptional regulation of the *ssl1* gene of CA-MRSA strain MW2, an *ssl1* promoter::lux fusion was constructed and transformed into *S.* *aureus* strains RN6390 and Newman. Resulting strains were grown in a defined minimal medium (DSM) broth and nutrient-rich brain-heart infusion (BHI) broth and expression was determined by luminescence. Transcription of *ssl1* was up-regulated and occurred earlier during growth in DSM broth compared to BHI broth suggesting expression is regulated by nutrient availability. RN6390 and Newman strains containing the *ssl1::lux* fusion were also used to analyze regulation in vivo using a mouse abscess model of infection. A marked increase in *ssl1* transcription occurred early during infection, suggesting *SSL1* is important during early stages of infection, perhaps to avoid the immune system.

## 1. Introduction

*Staphylococcus aureus* is one of the most clinically significant human pathogens and is responsible for a wide variety of infections, ranging from skin and soft tissue infections (e.g., boils, furuncles, etc.) to more invasive diseases (e.g., bacteremia, endocarditis, pneumonia, toxic shock syndrome, and necrotizing fasciitis) [1]. Presently, *S. aureus* causes close to 700,000 infections annually in the United States and is the leading cause of nosocomial infections in the United States [1]. Many healthcare facilities in the United States have endemic problems with methicillin-resistant *S. aureus* (MRSA), with ~60% *S. aureus* infections caused by MRSA strains [2]. In 1997, community-associated methicillin-resistant *S. aureus* (CA-MRSA) strains emerged in the United States, causing infections in people with no known risk factors [3] and have become the major cause of skin and soft tissue infection [2].

The disease-causing ability of CA-MRSA can be attributed to an impressive list of virulence factors. Many of these factors are shared with all *S. aureus* strains and include lipases, nucleases, proteases, hyaluronidase, collagenase, exfoliative toxins, leukocidins, and four hemolysins [4]. Along with these virulence factors, CA-MRSA strains have been shown to carry various novel putative toxin genes, including staphylococcal enterotoxin homologues (*seg*2, *sel*2, *sec*4, and *sek*2), *bsa* (a bacteriocin), *cna*, *ear*, and *lpl*10 [5,6]. Most of these novel toxins have yet to be fully characterized but may represent a repertoire of factors necessary for the disease-causing capability of MRSA in the community.

Another example of less understood *S. aureus* virulence factors is a family of toxin genes originally described as staphylococcal exotoxin-like (*set*) genes [7]. These genes have since been renamed staphylococcal superantigen-like (*ssl*) to reflect their structural similarity to the staphylococcal superantigens [8]. There is a total of fourteen *ssl* genes, of which at least a subset is found in nearly all *S. aureus* strains tested [5,6,7,9,10]. SSL proteins share the typical superantigen structure, including a characteristic oligonucleotide/oligosaccharide binding (OB-fold) domain linked to a beta-grasp domain [7,11,12]. However, while genuine superantigens are pyrogenic, increase the lethality of endotoxin up to 100,000-fold [4], and non-specifically stimulate T-cells by cross-linking major histocompatibility complex-II molecules with T-cell receptors [12], SSL proteins do not have any of these superantigen capabilities [13,14,15]. Rather, functional analysis of members of the SSL family has shown a link to immune avoidance. For example, SSL7 binds efficiently to IgA, which limits leukocyte activation, and also binds complement C5 protein preventing its cleavage into C5a and C5b, which reduces the proinflammatory activity [11,16,17]. Furthermore, other SSL proteins also play a role in immune avoidance by binding efficiently to phagocytic cells, preventing rolling adhesion of neutrophils and attachment of IgA [10,18]; by binding to tenascin, affecting cell motility of keratinocytes [19]; and by inhibiting toll-like receptor 2 [20,21].

SSL proteins are found exclusively in *S. aureus*, but not all *S. aureus* strains have the same array of SSL proteins [15]. Genome sequencing shows that the *ssl* genes share 36% to 67% homology [5,6,22]. Allelic differences of individual *ssl* genes among *S. aureus* strains show 81% to 95% homology at the nucleotide level and 77% to 94% homology at the amino acid level [15]. However, allelic differences have not been shown to affect the function of the SSLs. Transcription studies have shown that for most of the *ssl* genes there is up-regulated transcription during the stationary phase of growth in nutrient rich media [7,11,15]. Furthermore, SSL proteins are recognized by the human immune system as shown by seroconversion in human patients [14,15,23]. The binding of human antibodies to SSL proteins seems to be specific and not cross-reactive [23].

SSL proteins are likely to contribute to the virulence of MRSA in the community setting. Both dominating strains of CA-MRSA, MW2, a prototypical USA400 clone of CA-MRSA first described after an outbreak in North Dakota and Minnesota in 1998 [3], and USA300 clones, the other dominating CA-MRSA PFGE type [6,10,24] have all fourteen *ssl* genes. Furthermore, in a comparison of virulence genes found in commensal or clinical MSSA isolates versus CA-MRSA (MW2) isolates, it was found that 100% of the MW2 isolates tested were positive for the *ssl1* gene, which was significantly greater than the number of *ssl1* positive MSSA isolates [25]. The following study demonstrates that the expression of *ssl1* is enhanced in nutrient poor broth medium compared to nutrient rich medium, suggesting regulation of this gene responds to nutrient limitation that may be encountered within the host. Furthermore, we demonstrate that *ssl1* expression occurs early in a murine abscess model of infection, consistent with the hypothesis that the SSL proteins are important for immune avoidance.

## 2. Results

### 2.1. Expression of ssl1 is Up-Regulated in Nutrient-Poor Conditions

To examine the expression of *ssl1*, a vector containing an *ssl1::lux* promoter fusion was constructed in the shuttle plasmid pXen5 [26]. The *ssl1* promoter was amplified from genomic DNA of the *S. aureus* MW2 strain. Previous research has shown that most *ssl* genes, including *ssl1*, showed increased expression during the stationary phase [7,15]. However, previous reports of *ssl* gene expression were done in nutrient rich media. To test whether expression would also occur in nutrient poor defined minimal medium (DSM), the reporter fusion construct was transformed into *S. aureus* strain RN4220 (resulting in strain WS4108). This strain was inoculated into both DSM and brain-heart infusion (BHI) broth and examined for growth and luminescence. Initially, there was measurable luminescence following growth in both media, demonstrating that *ssl1* gene expression occurs under both nutrient poor and nutrient rich conditions and provided the rationale to test expression in virulent *S. aureus* strains (data not shown).

**Figure 1:**
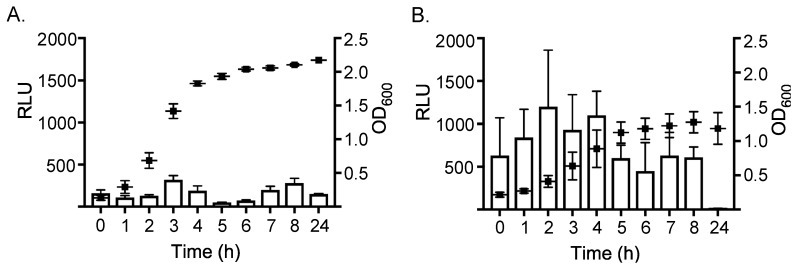
Expression of staphylococcal superantigen-like (*ssl1*) in strain WS0501 (RN6390 background) grown in (**A**) brain-heart infusion (BHI) broth or (**B**) defined minimal medium (DSM) broth. One milliliter aliquots were removed every hour for 8 h and at 24 h to measure luminescence (white column) and optical density (600 nm) (black diamond). Relative luminescence units (RLU) were divided by the viable colony forming units and multiplying the per cell luminescence by 10^6^ to obtain the final RLU shown. The data represents the RLU means + standard deviation of at least three trials.

**Figure 2:**
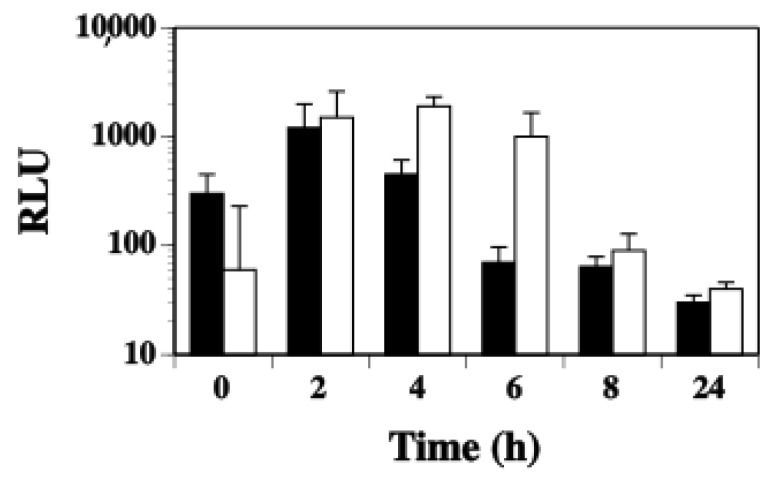
Expression of *ssl1* in strain NS3513 (Newman background) grown in BHI broth (black column) and DSM broth (white column). One milliliter aliquots were removed every 2 h for 8 h and at 24 h to measure luminescence. Luminescence (RLU) was determined as was presented before. The data represents the RLU means + standard deviation of at least three trials.

The *ssl1::lux* promoter fusion was subsequently integrated into the chromosome of *S. aureus* strain RN6390, resulting in strain WS0501. RN6390 has a genetically similar background as RN4220, but is more virulent and conducive for in vivo infection models [27]. Expression of *ssl1* was then assessed in this strain, and in all other experiments discussed below, by harvesting 1 ml of culture at given time points and normalizing the raw luminescence to total colony forming units resulting in the reported relative luminescence units (RLU). Absolute optical density was different, but the overall growth curve pattern was similar, between growth conditions. Despite any subtle differences in growth, all RLU values reflect the per cell expression when normalized to colony forming units. Expression of *ssl1* in the virulent WS0501 strain was markedly different depending on the growth environment. In nutrient rich broth, expression of *ssl1* was relatively low throughout the entirety of the growth curve (Figure 1A), with minor peaks of expression as the culture reached late log phase growth (320 RLU, OD_600_ = 1.418) and again during stationary phase (280 RLU, OD_600_ = 2.106). In contrast, in nutrient poor medium (Figure 1B), expression of *ssl1* was markedly higher, ranging from two (*p* < 0.05) to eleven-fold (*p* < 0.001) higher RLU per growth curve time point compared to the same time points during growth in BHI. For example, after two hours of growth in DSM expression of *ssl1* (1200 RLU) was 9.2-fold greater than expression at the same time point following growth in nutrient rich BHI (130 RLU) (*p* < 0.001). Moreover, peak expression of *ssl1* in DSM occurred during log phase growth and there was not a second peak during stationary phase, though expression remained higher at these time points relative to expression in nutrient rich broth. To determine if the expression of *ssl1* was strain-dependent we integrated the same *ssl1::lux* reporter fusion into *S. aureus* Newman. Expression of *ssl1* in this Newman strain construct (strain NS3513) was consistent with the *ssl1* expression demonstrated in the RN6390 background (Figure 2). For example, after 4 h of growth in DSM, expression was 3.25-fold higher (2700 RLU) compared to expression in BHI (830 RLU). Expression remained higher after 6 h in DSM, before both cultures reduced expression during late stationary phase. Thus, both strains showed higher *ssl1* transcription when grown in DSM broth compared to BHI broth.

### 2.2. Transcription of Other ssl Genes Is Up-Regulated in Nutrient Poor Conditions

**Figure 3:**
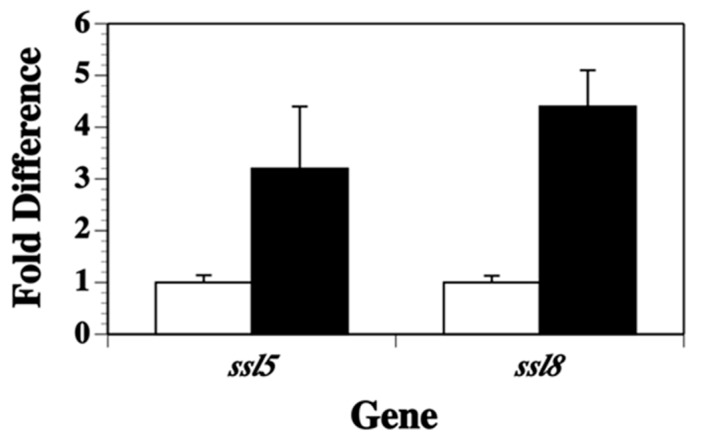
Quantitative reverse transcribed-polymerase chain reaction results of *S. aureus* strain RN6390 *ssl5* and *ssl8* transcription grown in BHI broth (white column) compared to DSM broth (black column). Transcription following growth in BHI broth was set as the baseline values. The data represents the mean + standard deviation from three separate runs.

The luminescence results indicated that *ssl1* transcription was elevated in *S. aureus* grown in a nutrient poor growth environment (DSM broth) compared to growth in a nutrient rich environment (BHI broth). To determine if this elevated transcription in nutrient poor conditions occurred for other *ssl* genes, a quantitative real time-polymerase chain reaction (qRT-PCR) procedure was used that targeted the *ssl5* and *ssl8* genes. Total RNA was collected at early stationary growth, when we observed high levels of *ssl1* expression. Consistent with the expression observed for *ssl1*, both *ssl5* (3.2-fold increase, *p* < 0.04) and *ssl8* transcript abundance (4.4-fold increase, *p* < 0.003) significantly increased in strain RN6390 grown in DSM broth versus BHI broth (Figure 3). Thus, nutrient deprivation elicits an increase in the transcription of several *ssl* genes and the *ssl* genes may share common mechanisms of regulation.

### 2.3. Agr Has a Minor Contribution to the Modulation of ssl1 Expression in S. aureus

**Figure 4:**
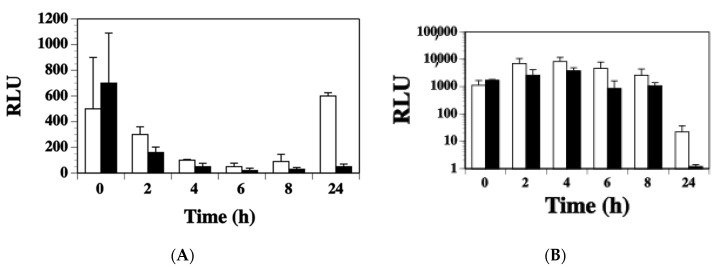
Expression of *ssl1* in strains WS0501 (RN6390 background, white column) and WS2601 (RN6390 agr mutant; black column) grown in (**A**) BHI broth or (**B**) DSM broth. One milliliter aliquots were removed every 2 h for 8 h and at 24 h to measure luminescence. RLU were determined as was presented before. The data represents the RLU means + standard deviation of at least three trials.

The accessory global regulator (*agr*) system of *S. aureus* controls the up-regulation of many toxin genes during stationary phase growth [28,29]. To test whether Agr was involved in regulating expression of *ssl1*, the *ssl1::lux* fusion was moved into the *agr* mutant strain RN6911 by transduction. Strains RN6911 and RN6390 are identical except for the *agr* mutation. Both strains were grown in BHI and DSM broth over a 24 h time period. In BHI broth, the *ssl1* expression was 2.3-fold lower in the *agr* mutant strain compared to the wild-type strain at the 2 h time point (Figure 4A, *p* = 0.06) and 2.7-fold lower in the *agr* mutant versus wild-type at 8 h (*p* = 0.099). After 24 h, the wild-type culture had a significantly higher transcription of *ssl1* compared to the *agr* mutant (12-fold, *p* = 0.046). When both strains were grown in DSM (Figure 4B), they had similar 0 h *ssl1* transcription levels (*agr* mutant 1700 RLU versus 1114 RLU for wild-type, *p* = 0.141). After 2 h of growth in DSM, the *agr* mutant showed 1.5-fold higher *ssl1* transcription and the wild-type strain 6.3-fold higher *ssl1* transcription versus their 0 h expression numbers, respectively. However, the *ssl1* expression increase was not significant when either comparing both strains at the 2 h reading or compared to their respective 0 h measurements. Both strains had the highest *ssl1* expression after 4 h in DSM broth (*agr* mutant 3759 and wild-type 8229). No significant difference was observed between the two strains at this time point (*p* = 0.197). The only significant difference in *ssl1* expression for the strains grown in DSM was also at the 24 h time point where the *ssl1* expression in the *agr* mutant was 1.2 RLU and in the wild-type it was 21.8 RLU (*p* = 0.036). Thus, *agr* may contribute to modulation of *ssl1* regulation, but not nearly to the extent that has been demonstrated for other toxins. Additionally, expression of *ssl1* still occurred in the absence of *agr* indicating additional unknown transcription factors regulate *ssl1*.

### 2.4. SaeS Positively Regulates ssl1 Expression in S. aureus

**Figure 5:**
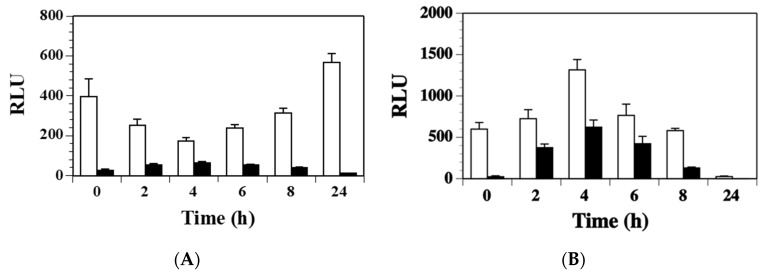
Expression of *ssl1* in strains WS0501 (RN6390 background, white column) and WS0604 (RN6390 *saeS* mutant; black column) grown in (**A**) BHI broth or (**B**) DSM broth. One milliliter aliquots were removed every 2 h for 8 h and at 24 h to measure luminescence. RLU were determined as was presented before. The data represents the RLU means + standard deviation of at least three trials.

Due to the observation that Agr did not greatly affect the regulation of *ssl1*, we sought to determine if another known regulatory system played a role. Other investigators have shown that SaeS positively regulates transcription of other *ssl* genes [30,31,32,33]. To examine whether SaeS positively regulated transcription of the *ssl1* gene, the *ssl1::lux* fusion was transduced into strain WS0604 that has an *saeS* mutation. At all time points examined, strain WS0604 displayed significantly lower *ssl1* expression (*p* < 0.05) compared to the wild-type strain when grown in both BHI broth (Figure 5A) and DSM broth (Figure 5B). Therefore, consistent with observations elsewhere, *ssl1* expression is positively regulated by SaeS in nutrient replete conditions, but also under nutrient poor conditions.

### 2.5. Expression of ssl1 Occurs Early in S. aureus Found in Murine Abscesses

**Figure 6:**
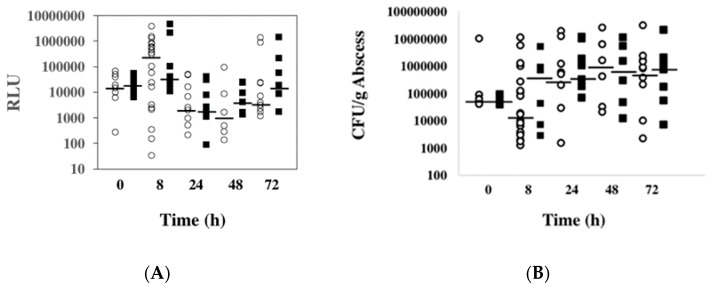
Expression of *ssl1* in a murine abscess model of infection. (**A**) Murine thighs were inoculated with either strain Newman (black column) or RN6390 (white column) and the thighs were collected at time points 0, 8, 24, 48, and 72 h post-inoculation. Each mouse is represented as the luminescence divided by the colony forming units (CFU) and then multiplied by 10^6^ (RLU). The black lines represent the median values of at least six mice for each strain per time point. (**B**) The CFU/g of abscess for each mouse infected with WS0501 (RN6390 background, white circles) or NS3513 (Newman background, black squares) at 0, 8, 24, 48, and 72-h post-inoculation. Black lines represent the median values for each strain at each time point.

To date, there is no direct evidence of any *ssl* gene expression in an animal model, although these proteins are produced during human infections indicated by antibody binding of human sera in reaction to these proteins [14,15,23]. Since CA-MRSA causes skin and soft tissue infections, *ssl1* expression in *S. aureus* growing in mammalian tissue was tested. Mouse thighs were inoculated with virulent strain WS0501 (*S. aureus* RN6390 background) followed by sacrifice of mice and collection of tissue at 0 h, 8 h, 24 h, 48 h, and 72 h post-infection. As was done in vitro, RLUs were normalized to viable colony forming units and the median values determined (Figure 6A,B). A baseline value at 0 h collected soon after inoculation showed 16,941 RLU for strain WS0501, indicating that the per cell expression of *ssl1* increased quickly in the host. After 8 h post-inoculation, this had increased further to 298,375 RLU, representing a 17.6-fold increase compared to the 0 h time point (*p* = 0.021). At 24 h post-inoculation, there was a significant decline to 2477 RLU, representing a 120.4-fold drop from the 8 h time point (*p* = 0.015). After 48 h post-inoculation, the expression decreased slightly to 1,061 RLU. Finally, after 72 h post-inoculation, the expression rose again to 5404 RLU, representing a 5.1-fold increase over the 48 h time point. We again wanted to minimize strain-specific effects on *ssl1* expression, so we repeated this experiment in the Newman strain background, strain NS3513. As was seen in vitro, *ssl1* expression was consistent between both strains in vivo. For example, there was a significant 1.94-fold increase in the median RLU value for *ssl1* transcription from 0 h to 8 h in the murine thighs (*p* = 0.016). There was also a second peak of expression later during infection at 72 h where the median RLU was 18,229.

For mice infected with the RN6390 strain background, the CFU/g abscess dipped when comparing the 0 h time point to the 8 h time point (Figure 4A). However, the colony numbers increased markedly at 24 h and reached their peak after 48 h post-inoculation. On the other hand, mice infected with the Newman strain background exhibited a more than 10-fold increase in CFU/g going from 0 h to 8 h, reaching a peak also at 48 h post-inoculation.

Collectively, this data demonstrated that there is robust *ssl1* expression during infection and abscess formation, with peak expression early during the infection when access to nutrients within the host tissue may be limited.

## 3. Discussion

In recent years, there has been an increase in *S. aureus* skin and soft tissue infections [1]. This increase of infections in community settings is certainly multifactorial and linked to the multitude of virulence factors encoded by *S. aureus*. Amongst these other virulence factors, CA-MRSA strains encode more of the *ssl* genes compared to the repertoire of other *S. aureus* strains, so the virulence of these strains may be influenced by the SSL proteins [3,6,10,24,25]. While the structure and corresponding functions of several SLL proteins have been described [19,20,34,35,36], there is less known about the regulation of the *ssl* genes. Previous work has demonstrated that there is up-regulated transcription of the *ssl* genes during stationary phase growth in nutrient rich media, consistent with other known staphylococcal toxins [7,15]. In this study, we investigated the regulation of the *ssl1* gene in *S. aureus* grown in a nutrient-rich broth (BHI) and a nutrient-poor broth (DSM), and in murine abscesses.

To study *ssl1* gene regulation, an *ssl1::lux* promoter fusion was constructed and moved into the chromosome of multiple *S. aureus* strains. Importantly, the promoter was constructed from *S. aureus* MW2, and thus reflects the expression of this gene from a CA-MRSA strain. A modest up-regulation of *ssl1* transcription was observed during the stationary phase growth in BHI broth, consistent with previous research elsewhere [7,15]. However, *ssl1* transcription was different in the nutrient poor, DSM broth with peak expression occurring during log phase growth. Furthermore, overall expression of *ssl1* during growth in DSM broth was higher throughout the entirety of the growth curve as compared to growth in BHI broth. We observed similar results for expression of *ssl5* and *ssl8* in independent qRT-PCR experiments. These results suggest expression of the *ssl* genes may share similar regulatory mechanisms.

While the growth phase may be a factor in nutrient rich media, limited nutrient availability likely plays a more important role in the up-regulation. Emphasizing this point, Agr seems to have a limited modulatory effect on *ssl1* expression. The *agr* (accessory global regulator) gene codes for a component of a quorum-sensing system [28,29] that up-regulates many secreted proteins during post-exponential phase growth, while simultaneously down-regulating cell-surface-expressed factors [37]. It has also been shown that *agr* mutants are attenuated, solidifying its place as an important regulator of virulence factors [38]. Some investigation into the role of Agr in regulation of the *ssl* genes has been done. For example, a previous study performed by Laughton et al. [39] demonstrated that Agr did not control *ssl11* expression. Using similar methods and identical parental strains of *S. aureus,* our results are largely consistent with those results. However, we did observe a small reduction of *ssl1* transcription in an *agr* mutant strain, albeit not to statistically significant levels, which suggests a possible positive, modulatory role in regulation of *ssl1*. Agr-independent expression of staphylococcal toxins is not without precedent. For example, both the staphylococcal enterotoxin A and J expression are independent of Agr [40,41]. Overall, it is likely that Agr and stationary phase-dependent quorum sensing does not regulate *ssl1* expression, rather it is the reduction of nutrients that is the signal to increase expression. The nutrient poor conditions in DSM broth may better mimic nutrient conditions present at the onset of a *S. aureus* infection in or on the human body [42].

Growth in minimal medium may trigger a stress response that would involve SaeRS or Rot (repressor of toxins) [39,40]. Several studies have shown Sae regulator-binding sites in the promoter regions of *ssl* genes [43,44]. The SaeRS two-component system (TCS) has also been shown to be important for *S. aureus* virulence using animal models of infection [30,45,46]. Moreover, the SaeRS TCS is important for avoidance of human neutrophil-mediated killing [46]. Rot and SaeR have been shown in strain Newman and other *S. aureus* strains to work synergistically to activate *ssl* promoters, including the *ssl1* promoter [30,31,32,47]. Transcriptional activation of *ssl1* in vivo (discussed below) by Rot and SaeR in *S. aureus* growing in murine thigh abscesses at an early time point could help to explain the immune cell avoidance reported by Voyich et al. [46]. In this study, we have shown that SaeS does indeed appear to be a positive regulator of *ssl1* transcription. A *saeS* mutation significantly reduced *ssl1* transcription in the RN6390 strain at all time points tested during growth in both nutrient rich and nutrient poor conditions. The role of SaeRS and Rot in the regulation of *ssl1* and other *ssl* genes during nutrient poor, other environmental conditions, and in vivo is the topic of future study.

Beyond the role of nutrients in regulation in vitro, our study demonstrates evidence for the expression of *ssl1* in *S. aureus* infected animals. In vivo regulation of the other *ssl* genes in vivo is unknown and requires further study. It is clear that some of the SSL proteins must be produced during human infections, demonstrated by human patient sera binding to at least one of the SSL proteins [15]. However, not all SSL proteins were recognized by the sera, including *SSL1*. Whether this lack of antibody binding to the SSL proteins is due to low expression levels in vivo or a result of the complex interaction between the bacterial proteins and the host immune system is not known. In our study, we show that *ssl1* is indeed expressed during infection of mammalian tissue. The highest *ssl1* expression was 8 h post-inoculation, followed by a subsequent decrease in *ssl1* expression at 24 and 48 h. This peak expression early during infection corresponds to a time when the bacteria are establishing infection and therefore correlates with the evidence that the SSL proteins are important for immune avoidance, such as binding IgA and C5 proteins or inhibiting phagocytic cells from reaching the site of infection [10,11,16,17,19,48,49,50]. In fact, more recent studies have shown that *SSL1* limits neutrophil chemotaxis and migration via matrix metalloprotease inhibition [35] and can cleave human recombinant cytokines [36]. Our data further suggests that once the infection is established, the *SSL1* protein may no longer be central for survival of the *S. aureus* cells and is significantly repressed. However, as the abscess matures, *ssl1* expression may again increase as there is a secondary need for the *SSL1* protein to sustain the infection at this late stage. Collectively, our *ssl1* expression data is consistent with the model that the SSL proteins are important for infection and that their expression may be regulated in part by the availability of nutrients within the host.

## 4. Conclusions

This study investigated transcriptional regulation of *S. aureus ssl1* following growth in vitro and in vivo in murine abscesses. The in vitro analysis demonstrated that *ssl1* transcription was higher in *S. aureus* growing in nutrient limited medium (DSM) compared to nutrient rich medium (BHI broth). Maximum transcription was during log-phase growth in nutrient poor conditions. Importantly, we have shown that the *ssl1* gene is also transcriptionally regulated in *S. aureus* growing within murine abscesses with peak expression early during infection (8 h), which correlates with the likely impairment of neutrophil function by *SSL1* early during an infection.

## 5. Materials and Methods

### 5.1. Bacterial Strains, Plasmids, and Growth Conditions

Various *Staphylococcus aureus* strains were used to assess *ssl1* expression under various conditions including RN4220, RN6390, RN6911, and Newman. Strain NE1296 (*saeS* mutation) was obtained from the Network on Antimicrobial Resistance in Staphylococcus aureus (NARSA) strain repository (Table 1), representing part of the Nebraska Transposon Mutant Library [51]. Luria broth or agar was used for growth of *E. coli.* All *S. aureus* strains were grown in either BHI media or in a DSM originally described by Rudin et al. [52], but modified later to better support the growth of *S. aureus* [53]. Antibiotics were used at the following concentrations: erythromycin, 300 μg/mL for *E. coli* and 5 μg/mL for *S. aureus*; kanamycin, 250 μg/mL. The pXen5 plasmid, containing an erythromycin resistance gene, an origin of replication for *E. coli,* a temperature-sensitive origin of replication for *S. aureus*, as well as a Tn*4100* transposon that contains a promotorless *lux* operon and a kanamycin resistance gene, was used for cloning [26].

### 5.2. Construction of the ssl1::lux Fusion

Genomic DNA from *S. aureus* strain MW2 grown to stationary phase in BHI at 37 °C was extracted with a commercial kit (Edge Biosystems, Gaithersburg, MD, USA) with a lysostaphin (Sigma-Aldritch, St. Louis., MO, USA) addition at the first step. Primers to amplify the promoter region were designed based on the MW2 genome [5]. The promoter DNA amplification was done with a PE9700 Thermal Cycler (Perkin Elmer, Wellesley, MA, USA) and *Taq* polymerase under the following conditions: 35 cycles, 94 °C for 30 s, 55 °C for 30 s, and 72 °C for 1 min. The forward primer was 5′-CACTGAATTCCCACTTCTGGAATACGTTTG-3′ and the reverse primer was 5′-CATTGGTACCACCTGTTGCTAACATTCCCA-3′. The forward primer included an EcoRI restriction site at the 5′ end and a KpnI restriction site at the 5′ end of the reverse primer. The *ssl1* promoter was cloned upstream of a promoterless *lux* operon in plasmid pXen5 [26]. Resulting *E. coli* transformants were screened for luciferase activity using a Femtomaster FB12 luminometer (Zylux Corporation, Maryville, TN, USA). Several clones exhibited luminescence and plasmid DNAs were extracted from clones that were positive for luminescence. One plasmid, named pXssl, was electroporated into electrocompetent *S. aureus* strain RN4220 as previously described [56]. Transformed *S. aureus* cells were selected on BHI agar containing erythromycin and kanamycin. Resulting clones were grown in overnight cultures and checked for luminescence activity. A successful clone was incubated at 42 °C to silence the temperature-sensitive origin of replication and allow the Tn*4100* transposon with the *ssl1::lux* fusion to move into the chromosome. A final clone, WS4108, was obtained based on a loss of erythromycin resistance, due to the movement of the transposon into the chromosome, and positive luminescence.

### 5.3. Transduction of S. aureus

The *ssl1::lux* fusion in strain WS4108 was transduced into strains RN6390, RN6911, and Newman and the *saeS*::mariner mutation was transduced into strain WS0501 [57]. Briefly, a staphylococcal bacteriophage Φ80α lysate was prepared using WS4108 cells. The phage lysate was then used to transduce the various staphylococcal strains, plating the transduced cells onto BHI containing kanamycin. Colonies that arose were grown overnight and examined for luminescence.

### 5.4. In vitro Testing of the ssl1::lux Fusion

The *S. aureus* strains containing the *ssl1::lux* fusion were grown overnight in BHI and an aliquot of each overnight culture was used to inoculate fresh media the next day for testing of *ssl1* expression by luminescence analysis. All strains were grown in both BHI and DSM broth with kanamycin at 37 °C with shaking. At given time intervals spanning the growth curve, a 1 mL aliquot was removed from the culture and tested for luminescence and optical density (600 nm) as described above. Viable colony forming units were obtained by serial dilution with aliquots plated onto BHI agar plates and incubated at 37 °C overnight. RLU were determined by dividing the raw luminescence units by the viable colony forming units. Because the per cell luminescence was low, all values were multiplied by 10^6^ to obtain the final RLU values.

### 5.5. RNA Extraction and Quantitative Reverse Transcribed-Polymerase Chain Reaction (qRT-PCR)

Total RNA was isolated from *S. aureus* strain RN6390 cells grown to early stationary phase in DSM or BHI broth using a High Pure RNA Isolation kit (Roche Diagnostics, Indianapolis, IN, USA) with an additional lysostaphin treatment step to help lyse the *S. aureus* cell walls and DNase I digestion. RNA samples were analyzed on a Nanodrop machine (Thermo Scientific, Waltham, MA, USA) to assess concentration and purity as well as run on 0.8% agarose gels to confirm concentration and integrities of the RNAs. The cDNAs were synthesized from 2 μg of total RNA using a First-Strand Synthesis kit (Life Technologies, Carlsbad, CA, USA) according to manufacturer’s instructions. All of the RT-qPCRs were performed using the iTaq Universal SYBR Supermix kit according to manufacturer’s instructions (BioRad, Hercules, CA, USA). Primers used in this study have been used in other studies: *ftsZ* [58], *ssl5* [33], and *ssl8* [33] and were synthesized by Integrated DNA Technologies (Coralville, IA, USA). A CFX96 machine (BioRad, Hercules, CA, USA) was used throughout the study. The *ftsZ* housekeeping gene was used as a standardization control. Each RT-qPCR run followed the minimum information for publication of quantitative real-time PCR experiments guidelines [59]. The RT-qPCRs were done at least three times under the following conditions: 94 °C, 20 s; 55 °C, 30 s; and 72 °C, 1 min for 35 cycles. The level of target gene transcripts in RN6390 cells was compared to the *ftsZ* gene. Crossover points for all genes were standardized to the crossover points for *ftsZ* in each sample using the 2^−ΔΔCT^ formula [60].

### 5.6. Murine Abscess Model of Infection

The Institutional Animal Care and Use Committee at the University of Wisconsin-La Crosse approved the animal handling protocol of this study (Protocol 4-07, approved on 7 May 2007). A previously used murine abscess model of infection was used to test in vivo expression of the *ssl1::lux* fusion in strains WS0501 and NS3513 [61]. Briefly, cultures were grown to mid-logarithmic phase, diluted to 2 × 10^7^ CFU/mL, and mixed 1:1 with Cytodex beads (Sigma, St. Louis., MO, USA). Female Swiss Webster mice (Harlan) that were 8 to 16 weeks old were injected intramuscularly with 50 μL of the mixture into the right posterior thigh in batches of four or five mice per time point repeated at least once. At 0, 8, 24, 48, and 72 h post-infection, mice were sacrificed, and the infected thigh muscle tissues collected. Each thigh tissue was homogenized in one mL of phosphate-buffered saline (PBS) and luminescence measured. Luminescence readings were normalized by subtracting the background RLU of an uninfected control and then dividing by the viable colony forming units. Any mice that did not demonstrate a successful infection indicated by bacterial counts below the detection limit (300 CFU) or below the background luminescence, were excluded from the analysis. The results represent the averages from at least six mice.

### 5.7. Statistical Analysis

The results of the *ssl1* expression in mice were analyzed by a one-way ANOVA. To further analyze the differences between the mean RLU of each time point, a least significant differences post hoc test was performed. For in vitro analysis in the growth media, Student’s *t*-tests were performed. *p*-values of <0.05 were considered significant.

## Figures and Tables

**Table 1:** Bacterial strains and plasmids.

Strain or Plasmid	Relevant Characteristics	Reference
Strains		
*E. coli*		
DH5α	General cloning strain	Invitrogen
*S. aureus*		
MW2	CA-MRSA USA400 virulent strain	[5]
RN4220	Transformation efficient strain	[27]
RN6390	Virulent strain	[27]
RN6911	Agr inactive, RN6390 with *agr::tetM*	[54]
Newman	Virulent strain	[55]
NE1296	JE2 strain with *saeS* mutation	[51]
WS4108	RN4220 *ssl1::lux*	This study
WS0501	RN6390 *ssl1::lux*	This study
WS0604	RN6390 *ssl1::lux*, *saeS::mariner*	This study
WS2601	RN6911 *ssl1::lux*	This study
NS3513	Newman *ssl1::lux*	This study
Plasmids		
pXen5	TS ^a^ origin, *Tn4001,* promoterless *lux*	[26]
pXssl	*ssl1::lux* on pXen5	This study

^a^ TS = Temperature-sensitive.

## References

[1] Suaya J.A., Mera R.M., Cassidy A., O’Hara P., Amrine-Madsen H., Burstin S., Miller L.G. (2014). Incidence and cost of hospitalizations associated with *Staphylococcus aureus* skin and soft tissue infections in the United States from 2001 to 2009. BMC Infect. Dis..

[2] Klein E.Y., Sun L., Smith D.L., Laxminarayan R. (2013). The changing epidemiology of methicillin-resistant *Staphylococcus aureus* in the United States: A national observational study. Am. J. Epidemiol..

[3] Centers for Disease Control and Prevention (1999). Four pediatric deaths from community-acquired methicillin-resistant *Staphylococcus aureus*–Minnesota and North Dakota, 1997-1999. Morbid. Mortal. Wkly. Rep..

[4] Dinges M.M., Orwin P.M., Schlievert P.M. (2000). Exotoxins of *Staphylococcus aureus*. Clin. Microbiol. Rev..

[5] Baba T., Takeuchi F., Kuroda M., Yuzawa H., Aoki K., Oguchi A., Nagai Y., Iwama N., Asano K., Naimi T. (2002). Genome and virulence determinant of high virulence community-acquired MRSA. Lancet.

[6] Diep B.A., Gill S.R., Chang R.G., Phan T.H., Chen J.H., Davidson M.G., Lin F., Lin J., Carleton H., Mongodin E.F. (2006). Complete genome sequence of USA300, an epidemic clone of community-acquired methicillin-resistant *Staphylococcus aureus*. Lancet.

[7] Williams R.J., Ward J.M., Henderson B., Poole S., O’Hara B.P., Wilson M., Nair S.P. (2000). Identification of a novel gene cluster encoding staphylococcal exotoxin-like proteins: Characterization of the prototypic gene and its gene product, SET1. Infect. Immun..

[8] Lina G., Bohach G.A., Nair S.P., Hiramatsu K., Jouvin-Marche E., Mariuzza R. (2004). Standard nomenclature for the superantigens expressed by *Staphylococcus*. J. Infect. Dis..

[9] Holtfreter S., Bauer K., Thomas D., Feig C., Lorenz V., Roschack K., Friebe K., Selleng S., Lovenich S., Greve T. (2004). *egc*-encoded superantigens from *Staphylococcus aureus* are neutralized by human sera much less efficiently than are classical staphylococcal exotoxins or toxic shock syndrome toxin. Infect. Immun..

[10] McCarthy A.J., Lindsay J.A. (2013). *Staphylococcus aureus* innate immune evasion is lineage-specific: A bioinformatics study. Infect. Genet. Evol..

[11] Langley R., Wines B., Willoughby N., Basu I., Proft T., Fraser J.D. (2005). The staphylococcal superantigen-like protein 7 binds IgA and complement C5 and inhibits IgA-Fc(alpha)RI binding and serum killing of bacteria. J. Immunol..

[12] Papageorgiou A.C., Achaya K.R. (2000). Microbial superantigens: From structure to function. Trends Microbiol..

[13] Al-Shangiti A.M., Naylor C.E., Nair S.P., Briggs D.C., Henderson B., Chain B.M. (2004). Structural relationships and cellular tropisms of staphylococcal superantigen-like proteins. Infect. Immun..

[14] Arcus V.L., Langley R., Proft R., Fraser J.D., Baker E.N. (2002). The three-dimensional structure of a superantigen-like protein, SET3, from a pathogenicity island of the *Staphylococcus aureus* genome. J. Biol. Chem..

[15] Fitzgerald J.R., Reid S.D., Ruotsalainen E., Tripp T.J., Liu M.Y., Cole R., Kuusela P., Schlievert P.M., Jarvinen A., Musser J.M. (2003). Genome diversification in *Staphylococcus aureus*: Molecular evolution of a highly variable chromosomal region encoding the staphylococcal exotoxin-like family of proteins. Infect. Immun..

[16] Lorenz N., Clow F., Radcliff F.J., Fraser J.D. (2013). Full functional activity of SSL7 requires binding of both complement C5 and IgA. Immunol. Cell Biol..

[17] Wines B.D., Ramsland P.A., Trist H.M., Gardam S., Brink R., Fraser J.D., Hogarth P.M. (2011). Interaction of human, rat, and mouse immunoglobin A (IgA) with staphylococcal superantigen-like 7 (SSL7) decoy protein and leukocyte IgA receptor. J. Biol. Chem..

[18] Hermans S.J., Baker H.M., Sequeira R.P., Langley R.J., Baker E.N., Fraser J.D. (2012). Structural and functional properties of staphylococcal superantigen-like protein 4. Infect. Immun..

[19] Itoh S., Yamaoka N., Kamoshida G., Takii T., Tsuji T., Hayashi H., Onozaki K. (2013). Staphylococcal superantigen-like protein 8 (SSL8) binds to tenascin C and inhibits tensacin C-fibronectin interaction and cell motility of keratinocytes. J. Biol. Chem..

[20] Koymans K.J., Feitsma L.J., Brondijk T.H., Aerts P.C., Lukkien E., Lossl P., van Kessel K.P., de Haas C.J., van Strijp J.A., Huizinga E.G. (2015). Structural basis for inhibition of TLR2 by staphylococcal superantigen-like protein 3 (SSL3). Proc. Natl. Acad. Sci. USA.

[21] Yokoyama R., Itoh S., Kamoshida G., Takii T., Fujii S., Tsuji T., Onozaki K. (2012). Staphylococcal superantigen-like protein 3 binds to the Toll-like receptor 2 extracellular domain and inhibits cytokine production induced by *Staphylococcus aureus*, cell wall component, or lipopeptides in murine macrophages. Infect. Immun..

[22] Kuroda M., Ohta T., Uchiyama I., Baba T., Yuzawa H., Kobayashi I., Cui L., Oguchi A., Aoki K., Nagai Y. (2001). Whole genome sequencing of methicillin-resistant *Staphylococcus aureus*. Lancet.

[23] Al-Shangiti A.M., Nair S.P., Chain B.M. (2005). The interaction between staphylococcal superantigen-like proteins and dendritic cells. Clin. Exp. Immun..

[24] Tenover F.C., McDougal L.K., Goering R.V., Killgore G., Projan S.J., Patel J.B., Dunman P.M. (2006). Characterization of a strain of community-associated methicillin-resistant *Staphylococcus aureus* widely disseminated in the Untied States. J. Clin. Microbiol..

[25] Shukla S.K., Karow M.E., Brady J.M., Stemper M.E., Kislow J., Moore N., Wroblewski K., Chyou P.H., Warshauer D.M., Reed K.D. (2010). Virulence genes and genotypic associations in nasal carriage, community-associated methicillin-susceptible and methicillin-resistant USA400 *Staphylococcus aureus* isolates. J. Clin. Microbiol..

[26] Francis K.P., Yu J., Bellinger-Kawahara C., Joh D., Hawkinson M.J., Xiao G., Purchio T.F., Caparon M.G., Lipitsch M., Contag P.R. (2001). Visualizing pneumococcal infections in the lungs of live mice using bioluminescent *Streptococcus pneumoniae* transformed with a novel gram-positive lux transposon. Infect. Immun..

[27] Novick R.R., Novick R.P. (1990). The *Staphylococcus* as a molecular genetic system. Molecular Biology of the Staphylococci.

[28] Recsei P., Kreiswirth B., O’Reilly M., Schlievert P., Gruss A., Novick R.P. (1986). Regulation of exoprotein gene expression in *Staphylococcus aureus* by *agr*. Mol. Gen. Genet..

[29] Yarwood J.M., Schlievert P.M. (2003). Quorum sensing in *Staphylococcus* infections. J. Clin. Investig..

[30] Nygaard T.K., Pallister K.B., Ruzevich P., Griffith S., Vunong C., Voyich J.M. (2010). SaeR binds a consensus sequence within virulence promoters to advance USA300 pathogenesis. J. Infect. Dis..

[31] Benson M.A., Lilo S., Wassaerman G.A., Thoendel M., Smith A., Horswill A.R., Fraser J., Novick R.P., Shopsin B., Torres V.J. (2011). *Staphylococcus aureus* regulates the expression and production of the staphylococcal superantigen-like secreted proteins in a Rot-dependent manner. Mol. Microbiol..

[32] Sun F., Li C., Jeong D., Sohn C., He C., Bae T. (2010). In the *Staphylococcus aureus* two-component sae, the response regulator SaeR binds to a direct repeat sequence and DNA binding requires phosphorylation by the sensor kinase SaeS. J. Bacteriol..

[33] Pantrangi M., Singh V.K., Wolz C., Shukla S.K. (2010). Staphylococcal superantigen-like genes, *ssl5* and *ssl8*, are positively regulated by Sae and negatively by Agr in the Newman strain. FEMS Microbiol. Lett..

[34] Dutta D., Dutta A., Bhattacharajee A., Basak A., Das A.K. (2014). Cloning, expression, crystallization and preliminary X-ray diffraction studies of staphylococcal superantigen-like protein 1 (*SSL1*). Acta Crystallogr. F Struct. Biol. Commun..

[35] Koymans K.J., Bisschop A., Vughs M.M., van Kessel K.P., de Haas C.J., van Strijp J.A. (2016). Staphylococcal superantigen-like protein 1 and 5 (*SSL1* & SSL5) limit neutrophil chemotaxis and migration through MMP-inhibition. Int. J. Mol. Sci..

[36] Tang A., Caballero A.R., Bierdeman M.A., Marquart M.E., Foster T.J., Monk I.R., O’Callaghan R.J. (2019). *Staphylococcus aureus* superantigen-like protein *SSL1*: A toxic protease. Pathogens.

[37] Dunman P.M., Murphy E., Haney S., Palacios D., Tucker-Kellogg G., Wu S., Brown E.L., Zagursky R.J., Shlaes D., Projan S.J. (2001). Transcription profiling-based identification of *Staphylococcus aureus* genes regulated by the *agr* and/or *sarA* loci. J. Bacteriol..

[38] Foster T.J., O’Reilly M., Phonimdaeng P., Cooney J., Patel A.H., Bramley A.J., Novick R.P. (1990). Genetic studies of virulence factors of *Staphylococcus aureus*. Properties of coagulase and gamma-toxin, alpha-toxin, beta-toxin and protein A in the pathogenesis of *S. aureus* infections. Molecular Biology of the Staphylococci.

[39] Laughton J.M., Devillard E., Heinrichs D.E., Reid G., McCormick J.K. (2006). Inhibition of expression of a staphylococcal superantigen-like protein by a soluble factor from *Lactobacillus reuteri*. Microbiology.

[40] Tremaine M.T., Brockman D.K., Betley M.J. (1993). Staphylococcal enterotoxin A gene (*sea*) expression is not affected by the accessory gene regulator (*agr*). Infect. Immun..

[41] Zhang S., Iandolo J.J., Stewart G.C. (1998). The enterotoxin D plasmid of *Staphylococcus aureus* encodes a second enterotoxin determinant (*sej*). FEMS Microbiol. Lett..

[42] Mekalanos J.J. (1992). Environmental signals controlling expression of virulence determinants in bacteria. J. Bacteriol..

[43] McNamara P.J., Milligan-Monroe K.C., Khalili S., Proctor R.A. (2000). Identification, cloning, and initial characterization of rot, a locus encoding a regulator of virulence factor expression in *Staphylococcus aureus*. J. Bacteriol..

[44] Giraudo A.T., Calzolari A., Cataldi A.A., Bogni C., Nagel R. (1999). The *sae* locus of *Staphylococcus aureus* encodes a two-component regulatory system. FEMS Microbiol. Lett..

[45] Montgomery C.P., Boyle-Vavra S., Daum R.S. (2010). Importance of the global regulators Agr and SaeRS in the pathogenesis of CA-MRSA USA300 infection. PLoS ONE.

[46] Voyich J.M., Vuong C., DeWald M., Nygaard T.K., Kocianova S., Griffith S., Jones J., Iverson C., Sturdevant D.E., Braughton K.R. (2009). The SaeR/S gene regulatory system is essential for innate immune evasion by *Staphylococcus aureus*. J. Infect. Dis..

[47] Benson M.A., Lilo S., Nygaard T., Voyich J.M., Torres V.J. (2012). Rot and SaeRS cooperate to activate expression of the staphylococcal superantigen-like exoproteins. J. Bacteriol..

[48] Besteboer J., Poppelier M.J.J.G., Ulfman L.H., Lenting P.J., Denis C.V., van Kessel K.P.M., van Strijp J.A.G., de Haas C.J.C. (2007). Staphylococcal superantigen-like 5 binds PSGL-1 and inhibits P-selectin-mediated neutrophil rolling. Blood.

[49] Chung M.C., Wines B.D., Baker H., Langley R.J., Baker E.N., Fraser J.D. (2007). The crystal structure of staphylococcal superantigen-like toxin 11 (SSL11) in complex with sialyl Lewis X reveals the mechanism for cell binding and immune inhibition. Mol. Microbiol..

[50] Swierstra J., Debets S., de Vogel C., Lemmens-den Toom N., Verkaik N., Ramdani-Bouguessa N., Jonkman M.F., van Dijl J.M., Fahal A., van Belkum A. (2015). IgG4 subclass-specific responses to *Staphylococcus aureus* antigens shed new light on host-pathogen interaction. Infect. Immun..

[51] Fey P.D., Endres J.L., Yajjala V.K., Widhelm T.J., Boissy R.J., Bose J.L., Bayles K.W. (2013). A genetic resource for rapid and comprehensive phenotype screening of nonessential *Staphylococcus aureus* genes. mBio.

[52] Rudin L., Sjostrom J.E., Lindberg M., Philipson L. (1974). Factors affecting competence for transformation in *Staphylococcus aureus*. J. Bacteriol..

[53] Schwan W.R., Wetzel K.J., Gomez T.S., Stiles M.A., Beitlich B.D., Grunwald S. (2004). Low-proline environments impair growth, proline transport and in vivo survival of *Staphylococcus aureus* strain-specific *putP* mutants. Microbiology.

[54] Novick R.P., Ross H.F., Projan S.J., Kornblum J., Kreiswirth B., Mohgazeh S. (1993). Synthesis of staphylococcal virulence factors is controlled by a regulatory RNA molecule. EMBO J..

[55] Duthie E.S., Lorenz L.L. (1952). Staphylococcal coagulase; mode of action and antigenicity. J. Gen. Microbiol..

[56] Iandolo J., Kraemer G.R. (1990). High frequency transformation of *Staphylococcus aureus* by electroporation. Curr. Microbiol..

[57] Kloos W.E., Pattee P.A. (1965). Transduction analysis of the histidine region in *Staphylococcus aureus*. J. Gen. Microbiol..

[58] Schwan W.R., Polanowski R., Dunman P.M., Medina-Bielski S., Lane M., Rott M., Lipker L., Wescott A., Monte A., Cook J.M. (2017). Identification of *Staphylococcus aureus* cellular pathways affected by the stilbenoid lead drug SK-03-92 using a microarray. Antibiotics.

[59] Bustin S.S., Benes V., Garson J.A., Hellemans J., Huggett J., Kubista M., Mueller R., Nolan T., Pfaffl M.W., Shipley G.L. (2009). The MIQE guidelines: Minimum information for publication of quantitative real-time PCR experiments. Clin. Chem..

[60] Livak K.J., Schmittgen T.D. (2001). Analysis of relative gene expression data using real-time quantitative PCR and the 2^−ΔΔCT^ method. Methods.

[61] Beonton B.M., Zhang J.P., Pope C., Christian T., Lee L., Winterberg K.M., Schmid M.B., Buysse J.M. (2004). Large-scale identification of genes required for full virulence of *Staphylococcus aureus*. J. Bacteriol..

